# Dr. Rhind on a New Remedy for Scalds and Burns

**Published:** 1842-11-12

**Authors:** William Rhind

**Affiliations:** Surgeon, Edinburgh


*On a New Remedy for Scalds and Burns.* By William Rhind, Surgeon, Edinburgh.—There are so many circumstances which lead one to be deceived in the operation of a new remedy, that I would with considerable diffidence have called the attention of the profession to the following facts, had not a lapse of a good many years, and repeated trials and strict observation confirmed me of their accuracy.

As in most other cases of remedial substances, a mere accidental circumstance first directed my attention to this discovery. In the year 1828, having let fall a drop of melted sealing-wax on the point of the finger, by which it was rendered extremely painful, I plunged it into a solution of gum-arabic, this being the only fluid substance at hand. In a few minutes I was surprised to find that the pain had completely ceased. I allowed this gum to harden on the part, and on washing it off several hours afterwards, I found the cuticle hardened and separated from the cutis, indicating the usual severity of the burn, but there was no blister or effusion of serum, and no pain or injury of the cutis. From this circumstance I resolved to try the farther effects of solution of gum-arabic in burns, and the following cases, which I find in my note-book, occurred some time after.

1828, April 9. A boy (aged 12) called on me at nine o'clock, evening. He had been firing off crackers of gunpowder, one of which had exploded and severely scorched the palms of both his hands and his fingers. The parts were red with excessive pain, and on one hand were three or four small blisters; these I opened, and pressed out the serum, and then with a large camel hair pencil smeared both hands over with a thick solution of gum-arabic. I went over the parts several times with this solution, so as to fix it completely over the surface of the skin, and then sent the boy out into the cool air. He returned in half-an hour. The pain of one hand was quite gone, and the other much easier. The solution was repeated till it thickly incrusted the hand, and he was again desired to walk about in the cool air, and to avoid hanging or moving the hand. In a very short time he again returned with the hand completely relieved of pain except in one spot, between the fingers, where the solution had not been applied. Here there was a small blister, which was immediately snipped, and after pressing out the serum the part was covered with the solution. At ten o'clock both hands were quite easy, and he expressed much satisfaction. During the night he felt no pain or unusual heat in his hands. Next morning he washed off the thick crust of gum, and when I saw him the cuticle of his palms was quite hard, and had all the appearanc of a severe burn, but no blisters had risen, and the few that had been opened discharged a little serum, but healed without giving any farther trouble.

1828, November 8. A workman employed at a brass-foundry got his face very much scalded by a quantity of hot lead or solder falling on it. I saw him a few minues after the accident. Large patches about his eyelids, cheeks, forehead, and lower lip, both outside and inside, were very much reddened, and were apparently beginning to rise into blisters. He complained of intense hot pain. Portions of the melted solder were picked from his lips, eyelids, and nose, and a solution of gum-arabic was applied immediately the whole extent of the injured parts. Repeated coats or coverings of this solution were applied at intervals of about five minutes till the whole began to harden over the face. He was desired to go home and keep himself in a cool place, and, if the pain did not abate in less than half-an hour, to return. I saw him in an hour, the pain had so completely left him that he was at dinner. No blister rose in this instance. A slight discharge occurred in two or three spots where the skin had been destroyed, but these healed in a very short time.

In my notes I find several other cases of burns and scalds in subsequent years, treated in a similar manner and with a like success, the details of which, however, would not afford any interest. In some of these cases an hour or more had elapsed since the accident, and oils and other applications had been used. In such instances, the skin was cleaned of the oily matters, by sponging it with soap and water, and then the solution was applied. In every case relief was procured in a very short time. The more recent the case, however, the more speedy was the removal of the pain. In those cases where blisters had appeared they were opened, and the solution was then applied; very frequently the application of the solution prevented the effusion of more serum; in some cases, however, serum was again effused and again evacuated. The last case which I have on record was 1840, March. Mrs ———, in reading too near to a candle, set fire to her gown at the shoulder, the thin fabric consumed immediately and severely scorched her arm. I saw her about ten minutes after. The whole shoulder and arm, embracing the space over the deltoid muscle and the greater part of the inside arm, were severely burnt, and she felt great pain. A thin solution of gum-arabic was immediately applied, while a thicker one was preparing, and several coats of this latter were spread over the parts repeatedly in the course of half-an hour. An hour after the accident the pain was almost entirely gone. A few blisters had risen about the inside of the arm; these were snipped, and the solution repeated. Next day, this patient had experienced no farther uneasiness from the arm, but the agitation and fright had brought on labour nearly about the full time. She was delivered of a still-born child, and had a good recovery. The blistered parts of the arm skinned off when the solution was washed away; the abraded parts were sprinkled with a little flour, and healed in a few days.

I have not had an opportunity of treating any cases of burning where there was much destruction of skin or soft parts, and therefore cannot say what effect the solution would have on those. I would suggest, however, that, if applied in such cases, a thin layer of raw cotton should either be smeared with the solution, or applied immediately after the fluid has been brushed over the parts, in order to form a sort of substitute for the skin, and a means of retaining the gum-arabic solution.

In those distressing cases of the extensive burning of the bodies of young children, I would not hesitate applying the solution over the whole body, at about the warmth of 96°. It does not cool down the system by sudden evaporation or sudden abstraction of heat like a common cold fluid, a circumstance in most cases to be dreaded, for gum is a bad conductor of heat; neither does it preclude an exposure to moderately cool air, which seems to keep down the excessive irritation consequent upon extensive scalding of the skin.

As it is of consequence to have the solution prepared instantly, the powdered gum, if it can be procured, may be in a few minutes dissolved in warm water. If this is not ready prepared, the common gum in small particles roughly pounded, will very soon dissolve, and the application in any case may be applied at a temperature of 96° or 100°, although in general it is more soothing when applied colder. Rancid gum solution should not be used, as it in this state has lost its adhesive quality. Two, three, or four applications may be necessary at intervals of five or ten minutes. The skin should be previously freed of all oily matters, and the first coating, in order that it may be insinuated closely into the furrowed surfaces of the skin, should be rather thinner than the subsequent ones. In order to produce the proper effect it should form a varnished coat of some thickness and closeness over the whole space of the burnt part.

With regard to the *modus operandi* of this substance, I am as much in the dark as we usually are with the mode of action of most well known remedies. I am unable to say whether it has a specific action, or whether it allays the inflammable irritation of the skin, by effectually excluding the external air, and by its being a bad conductor of heat.

The first proposition might be so far tested by trying if other gums and mucilages, or isinglass, for example, produced a similar effect. This I have not tried.

From some experiments, which, however, we never completed, that I instituted some years ago, regarding the influence of oxygen on inflamed tissues and vessels, I am inclined to think that the exclusion of atmospheric air influences very much inflammatory action, and in this way, perhaps, the gum solution checks the inflammation of the skin in burns. Inflammation caused by touching the skin with nitrous acid and other irritants, appears to be suddenly allayed by a solution of gum-arabic : erysipelatous spots on the skin seemed almost in some trials influenced by this application. And I may here suggest, that it might be tried in the first stages of the pustules of small-pox, especially those of the face, with a view to modify their development, and prevent pitting.

In conclusion, I deem it proper to mention, that, several years ago, I communicated the effects of this remedy to friends, both medical and others, and that several cases have been treated according to the above directions with uniform success. A few of those cases I find noted, but I do not perceive that they present any farther facts worthy of detail.—*Ibid.* Oct. 1, 1842.

